# Exploring ischemic stroke based on the ferroptosis perspective: ECH1 may serve as a new biomarker and therapeutic target

**DOI:** 10.3389/fnins.2025.1622760

**Published:** 2025-08-22

**Authors:** Rendong Qu, Yiyan Zhang, Haojia Zhang, Ke Li, Boning Zhang, Hongxuan Tong, Tao Lu

**Affiliations:** ^1^School of Life Sciences, Beijing University of Chinese Medicine, Beijing, China; ^2^Institute of Basic Theory for Chinese Medicine, China Academy of Chinese Medical Sciences, Beijing, China

**Keywords:** ischemic stroke, ferroptosis, peripheral blood, ECH1, tMCAO

## Abstract

**Background:**

Ischemic stroke (IS), the leading stroke subtype (∼87%), arises from vascular occlusions, triggering brain necrosis through ischemia-reperfusion injury. Ferroptosis, an iron-driven cell death via Fe^2+^-mediated lipid peroxidation, is implicated in IS pathology. This study demonstrates that enoyl-coA hydrolase 1 (ECH1) may serve as a peripheral biomarker and therapeutic target for IS based on ferroptosis signaling.

**Methods:**

We integrated transcriptome data from the GEO database with preprocessing and normalization. Hub genes were screened using differential expression analysis and machine learning algorithms. Subsequently, genes were further filtered by mendelian randomization and also validated using transient middle cerebral artery occlusion (tMCAO) model.

**Results:**

Ferroptosis-related genes in the IS group showed higher expression compared with the healthy control group. Using differential expression analysis and machine learning algorithms, 12 potential hub genes were successfully screened. Mendelian randomization analysis further confirmed the causal association between ECH1 and stroke. In the tMCAO mouse model, ECH1 mRNA levels were down-regulated, consistent with the results of the clinical samples.

**Conclusion:**

In this study, taking ferroptosis as an entry point, ECH1 may serve as a potential peripheral blood biomarker and therapeutic target for IS through multidimensional validation, providing a basis for the development of relevant precision diagnostic strategies.

## 1 Introduction

Ischemic stroke (IS), accounting for approximately 87% of all strokes, is mainly caused by atherothrombotic occlusion (e.g., carotid artery plaques) or cardioembolic occlusion (e.g., atrial fibrillation thrombi) (Martin et al., 2025). These thrombi can break off and block arteries, leading to insufficient blood supply and subsequent brain tissue necrosis. Emerging evidence highlights a close relationship between ferroptosis and stroke damage, with ferroptosis playing a pivotal role in the pathophysiology of IS (Gowtham et al., 2024; Liu et al., 2024; Tuo and Lei, 2024). Ferroptosis, a novel form of iron-metabolism-related cell death first proposed by Brent Stockwell in 2012, is characterized by the reaction of Fe^2+^ with hydrogen peroxide to generate Fe^3 +^, triggering lipid peroxidation and ultimately causing cell death (Dixon et al., 2012). In the context of IS, ischemia-reperfusion injury can elevate intracellular iron levels, activating ferroptosis pathways and exacerbating neuronal damage. Markers of ferroptosis, such as decreased expression of glutathione peroxidase 4 (GPX4) and the accumulation of lipid peroxides, serve as critical indicators of the severity of brain damage (Chai et al., 2024; Hu et al., 2024; Zhang et al., 2021). Previous studies have demonstrated that iron chelators and ferroptosis inhibitors (e.g., ferrostatin-1 and liproxstatin-1) can reduce brain damage and improve neurological function in animal models (Wang et al., 2023).

Given the current research landscape, the mechanisms and clinical applications of ferroptosis in IS warrant further exploration. Peripheral blood ferroptosis markers correlate with stroke severity, infarct volume, and neurological deficit scores, holding promise as diagnostic and prognostic biomarkers (Fan et al., 2025; Liu C. P. et al., 2025; Lu et al., 2020; Yeh et al., 2023; Zhang et al., 2022). Moreover, the relationship between ferroptosis and immune cells is gradually being uncovered, with peripheral blood immune cell changes following IS potentially reflecting immune mechanisms in the brain injury region (He et al., 2024).

In this study, building on the work of Zhang et al. (2022), we screened the core genes related to ferroptosis by differential expression analysis and machine learning algorithms. Subsequently, Mendelian randomization identified a causal relationship between ECH1 and stroke. We validated this result using C57BL/6J mice subjected to tMCAO modeling and also observed downregulation of the ECH1 mRNA levels in the peripheral blood and brain regions of the IS group, providing potential peripheral blood biomarkers and therapeutic targets for IS.

## 2 Results

### 2.1 Up-regulation of ferroptosis signals in ischemic stroke peripheral blood transcriptome

To determine whether peripheral blood ferroptosis signaling can serve as a biomarker for diagnosis and targeted therapy in IS patients, this study integrated publicly available human-derived gene expression datasets from the GEO database, containing 108 IS samples and 47 healthy control samples. After normalization (Supplementary Figures 1A–D), we performed batch effect correction between datasets using surrogate variable analysis (SVA) to eliminate technical biases that might affect subsequent analyses. To visualize the effect of batch correction, we generated principal component analysis (PCA) plots comparing the distribution of samples before and after batch correction (Supplementary Figures 1E, F).

**FIGURE 1:**
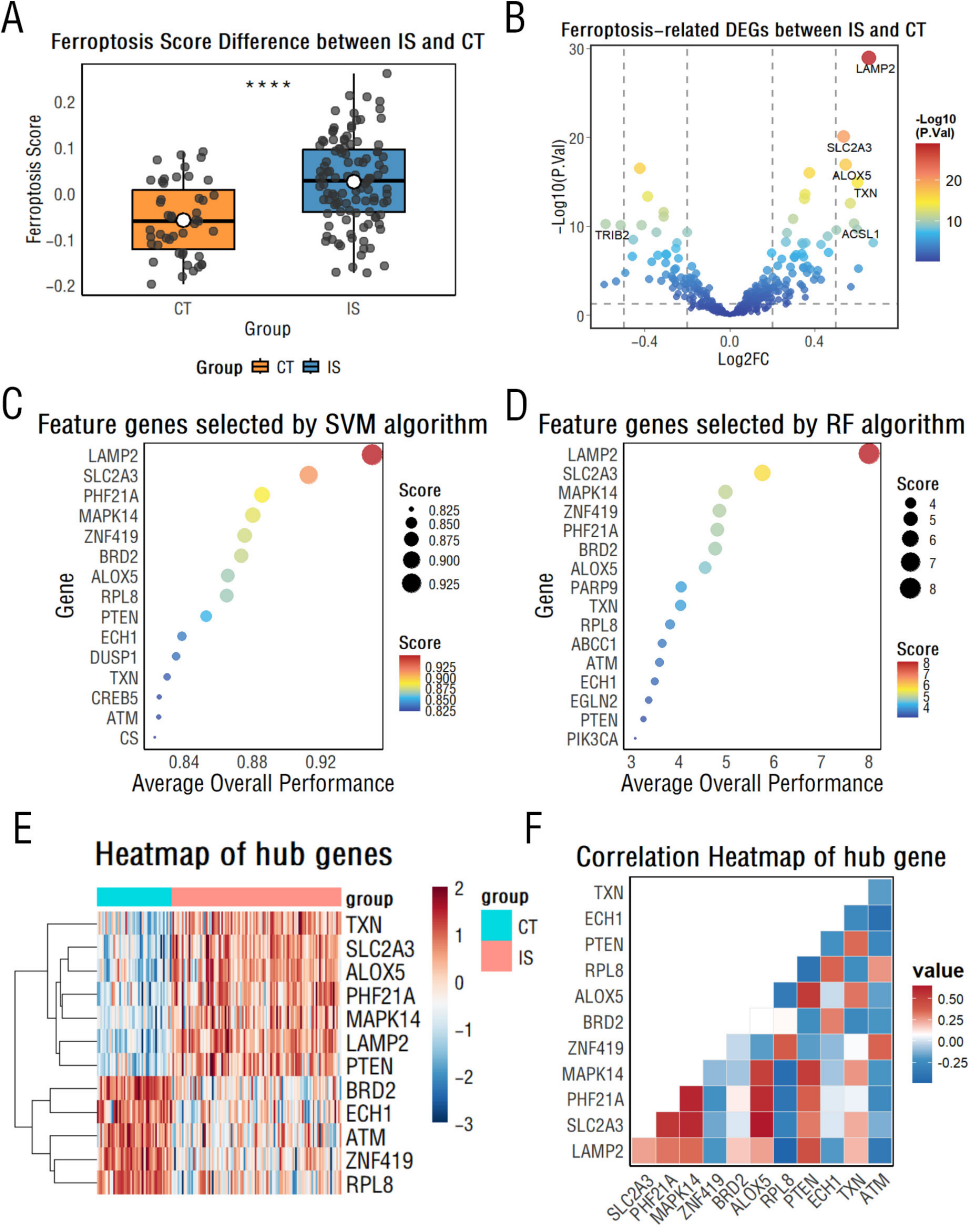
Ferroptosis-related gene analysis between CT and ischemic stroke (IS) groups. **(A)** Box plots display differences in Single Sample Gene Set Enrichment Analysis (ssGSEA) scores of ferroptosis-related genes between the CT and IS groups. ****p < 0.0001. **(B)** A volcano plot illustrates differentially expressed ferroptosis-related genes between the CT and IS groups. **(C,D)** Support vector machine **(C)** and random forest algorithms **(D)** rank the importance of ferroptosis-related genes, highlighting the top 15 key genes. **(E)** A heatmap visualizes key differentially expressed genes between the CT and IS groups. **(F)** A correlation heatmap reveals relationships among key ferroptosis-related genes.

Through the FerrDb database, we retrieved 565 ferroptosis-related genes, of which 410 genes were in the integrated dataset. Wilcoxon test with Benjamini-Hochberg (BH) correction was utilized to reveal that the expression of ferroptosis-related genes in the IS group was significantly higher than that in the healthy control group (adj.*P* < 0.05), suggesting that ferroptosis signaling is closely associated with IS injury (Figure 1A).

### 2.2 Identification and validation of hub genes in ischemic stroke by support vector machine and random forest algorithms

First, ferroptosis-related differentially expressed genes between the IS and healthy control groups were further identified, with a total of 178 differentially expressed genes, including 111 up-regulated genes and 67 down-regulated genes (Figure 1B).

Then, we employed support vector machine (SVM) and random forest (RF) algorithms with recursive feature elimination (RFE) to identify diagnostic biomarkers for IS. Model optimization was performed using 10-fold cross-validation, systematically evaluating performance across 1–20 feature genes. The optimal SVM model achieved 94.5% accuracy (SD = 0.076) with 15 features, while the RF model peaked at 93.5% accuracy (SD = 0.078) with 16 feature genes (Figures 1C, D and Supplementary Figures 2A, B). The intersection of selected feature genes from both algorithms yielded 12 hub genes: LAMP2, SLC2A3, PHF21A, MAPK14, ZNF419, ALOX5, RPL8, PTEN, BRD2, ECH1, TXN, and ATM. For these screened hub genes, their diagnostic performance in the training cohort was evaluated using the area under the receiver operating characteristic curve (AUC-ROC), with all exhibiting high AUC values greater than 0.75 (Supplementary Figure 2C).

To further validate their diagnostic robustness, we assessed these hub genes in the independent test cohort. All 12 hub genes maintained AUC values above 0.75 (Supplementary Figure 2D), confirming their stable ability to discriminate between the IS and healthy control groups. Meanwhile, as shown in Figure 1E, there was a statistically significant difference in the expression of these genes between the two groups (*P* < 0.05). In the IS group, ALOX5 showed the highest positive correlation with SLC2A3, while BRD2 showed the strongest negative correlation with TXN (Figure 1F).

### 2.3 Analysis of immune infiltration in ischemic stroke

**FIGURE 2:**
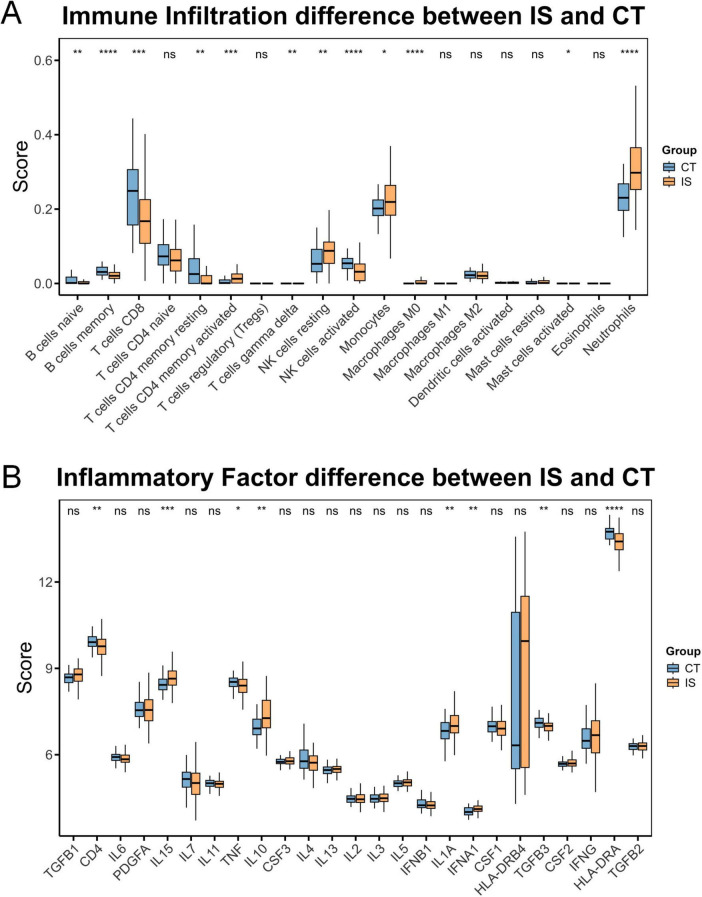
Immune profile differences between CT and ischemic stroke (IS) groups. **(A)** Differences in immune cell composition between the CT group and the IS group. **(B)** Differences in inflammatory factor levels between the CT group and the IS group. ns, not significant, *p < 0.05, **p < 0.01, ***p < 0.001, ****p < 0.0001.

**FIGURE 3:**
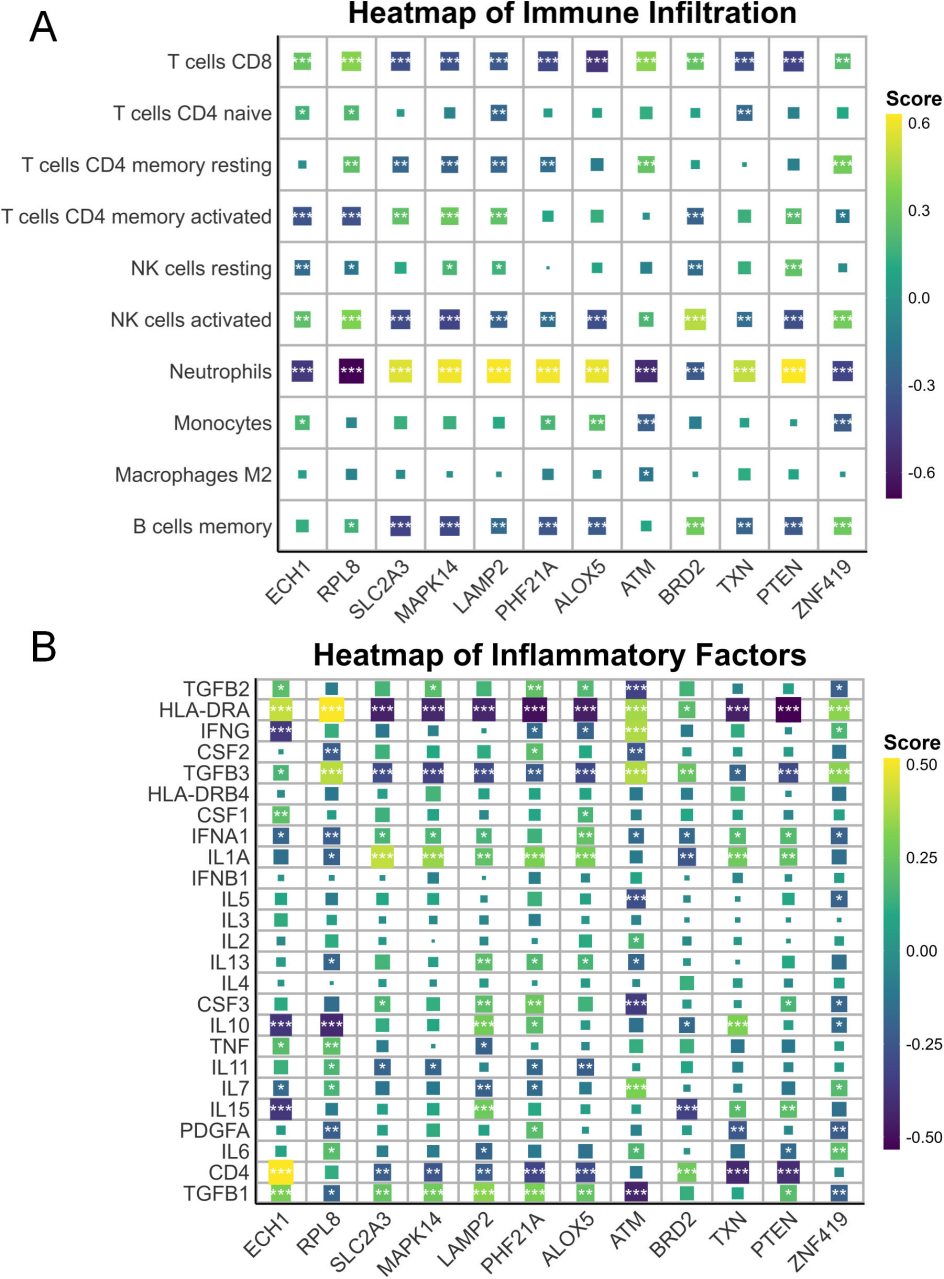
Immune and inflammatory correlations with ferroptosis genes. **(A)** Heatmap illustrating the associations between immunocytes and key ferroptosis-related genes. **(B)** Heatmap illustrating the associations between inflammatory mediators and key ferroptosis-related genes. *p < 0.05, **p < 0.01, ***p < 0.001.

It has been known that ferroptosis is strongly associated with immune cells, so we further carried out immune infiltration analysis (Liu C. et al., 2025; Zeng et al., 2024). The results showed that in the IS group, memory B cells, CD8+ T cells, resting CD4+ memory T cells, and active natural killer cells were relatively low, while active CD4+ memory T cells, resting natural killer cells, monocytes, and neutrophils were relatively high (Figure 2A). Additionally, CD4, HLA-DRA, TGFB3, and TNF were relatively low in the IS group, whereas IFNA1, IL10, IL15, and IL1A were relatively high (Figure 2B). These results are highly correlated with the hub ferroptosis genes described above (Figures 3A, B).

### 2.4 Cluster analysis shows ferroptosis signaling in ischemic stroke patients is not affected by age, sex, or short-term time after onset

**FIGURE 4:**
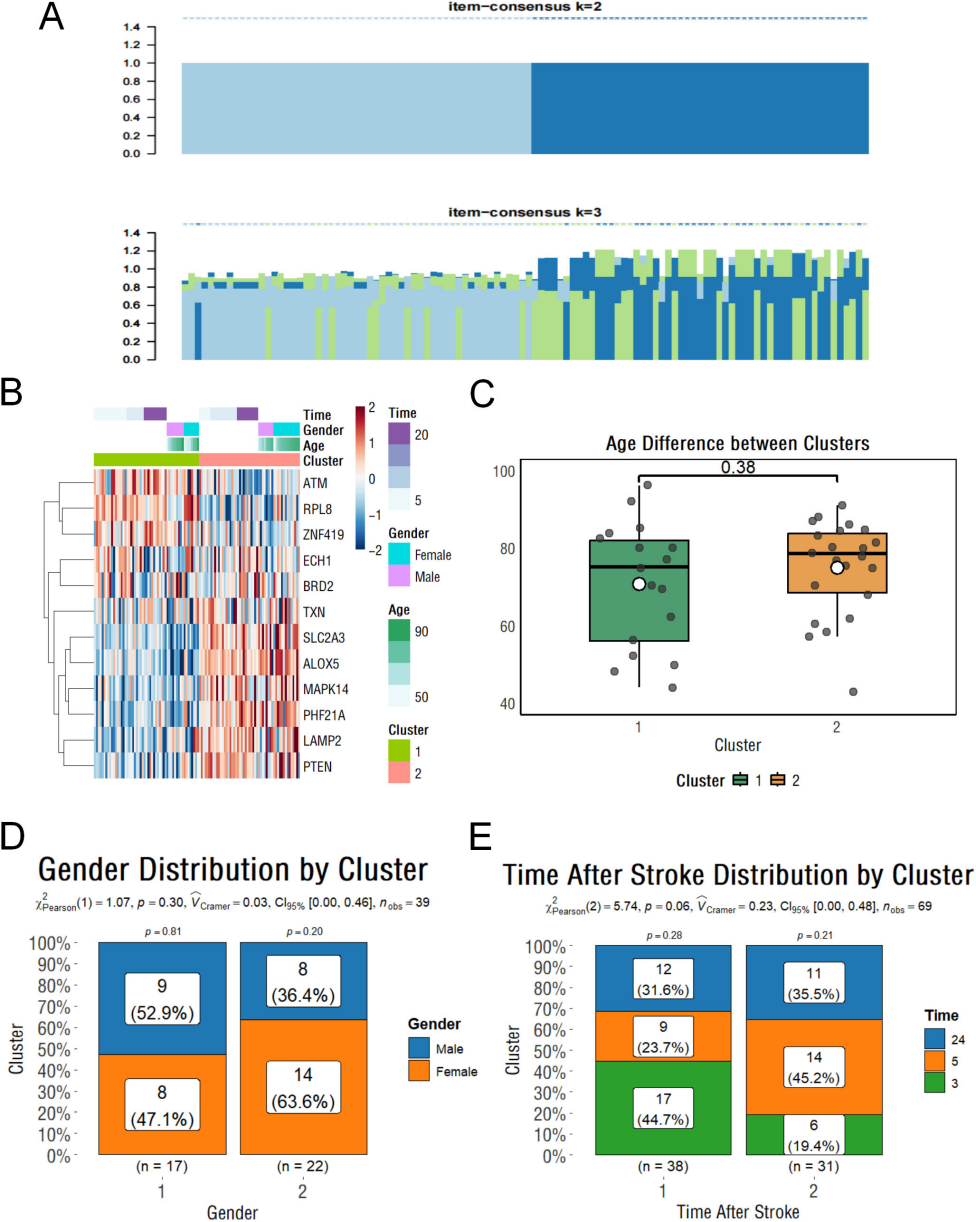
Clustering and clinical characteristics of stroke samples based on ferroptosis-related genes. **(A)** Clustering classification results of stroke samples based on key ferroptosis-related genes. **(B)** Heatmap showing gene expression levels with associated clinical information. **(C)** Boxplot showing the age differences between clusters. **(D)** Histogram illustrating the gender distribution within subgroups. **(E)** Histogram showing the distribution of stroke onset times across subgroups.

To determine whether the extent of ferroptosis in IS patients was influenced by other factors such as sex and age, we performed a cluster analysis based on the 13 hub genes described above, dividing these samples into two groups (Figure 4A). Our results indicate relatively strong ferroptosis signaling in subgroup 2. Genes like TXN, LAMP2, MAPK14, PTEN, PHF21A, and ALOX5 show higher expression than in subgroup 1 (Figure 4B). Subsequent analyses showed that there were no statistically significant differences in age, gender, or time of onset between the two groups, demonstrating the stability of these genes as biomarkers (Figures 4C–E).

### 2.5 Mendelian randomization further reveals that decreased peripheral blood ECH1 expression raises stroke risk

**FIGURE 5:**
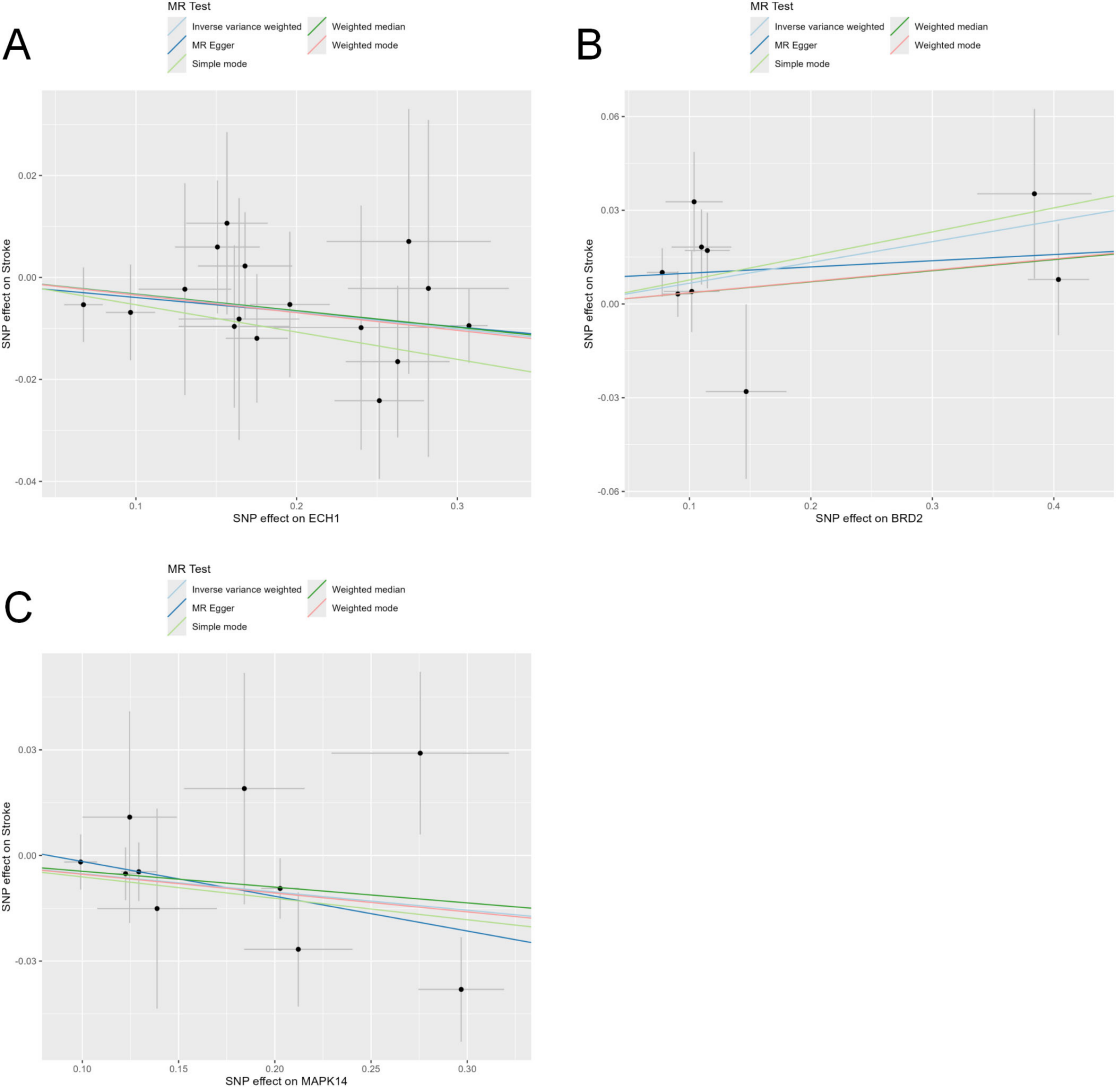
Mendelian randomization tests core genes causally linked to stroke. **(A–C)** Five Mendelian randomization methods, including the inverse-variance weighted (IVW), MR-Egger regression, weighted median, weighted mode, and simple mode, were employed to evaluate the causal relationship between stroke risk and genetic variants of the enoyl-coA hydrolase 1 (ECH1) **(A)**, BRD2 **(B)**, and MAPK14 **(C)**.

To further explore potential causal associations between these genes and stroke, we performed MR analysis on the exposure (Except for LAMP2 and SLC2A3, 10 of the 12 core genes could be retrieved) and outcome (stroke). Our results showed that ECH1, MAPK14, and BRD2 were significantly correlated with stroke, and no pleiotropy or heterogeneity was detected (*P* < 0.05) (Figures 5A–C and Table 1). The results of the leave-one-out analysis and single-SNP analysis are presented in the Supplementary Figures 4, 5. Notably, ECH1 showed a negative estimate effect in the MR results, indicating a relationship between decreased expression and increased Stroke risk, which showed a high consistency with the transcriptome results (ECH1 only; the other two genes were excluded because of the opposite result).

**TABLE 1:** Results from MR analysis of exposure (screened core genes) and outcome (stroke).

Exposure	Outcome	Method	NSNP	*P*-value	OR (95% CI)
ECH1	Stroke	Inverse variance weighted	16	0.037	0.967 (0.937 to 0.998)
ECH1	Stroke	MR Egger	16	0.453	0.972 (0.903 to 1.045)
ECH1	Stroke	Weighted median	16	0.116	0.968 (0.929 to 1.008)
ECH1	Stroke	Simple mode	16	0.163	0.948 (0.882 to 1.018)
ECH1	Stroke	Weighted mode	16	0.16	0.966 (0.923 to 1.011)
MAPK14	Stroke	Inverse variance weighted	10	0.02	0.950 (0.909 to 0.992)
MAPK14	Stroke	MR Egger	10	0.162	0.906 (0.799 to 1.027)
MAPK14	Stroke	Weighted median	10	0.13	0.956 (0.902 to 1.013)
MAPK14	Stroke	Simple mode	10	0.172	0.941 (0.868 to 1.020)
MAPK14	Stroke	Weighted mode	10	0.138	0.948 (0.889 to 1.011)
BRD2	Stroke	Inverse variance weighted	9	0.018	1.069 (1.012 to 1.129)
BRD2	Stroke	MR Egger	9	0.704	1.020 (0.925 to 1.125)
BRD2	Stroke	Weighted median	9	0.328	1.036 (0.965 to 1.113)
BRD2	Stroke	Simple mode	9	0.246	1.080 (0.957 to 1.218)
BRD2	Stroke	Weighted mode	9	0.387	1.037 (0.960 to 1.120)

### 2.6 ECH1 is relatively lowly expressed in peripheral blood in the tMCAO model mice

**FIGURE 6:**
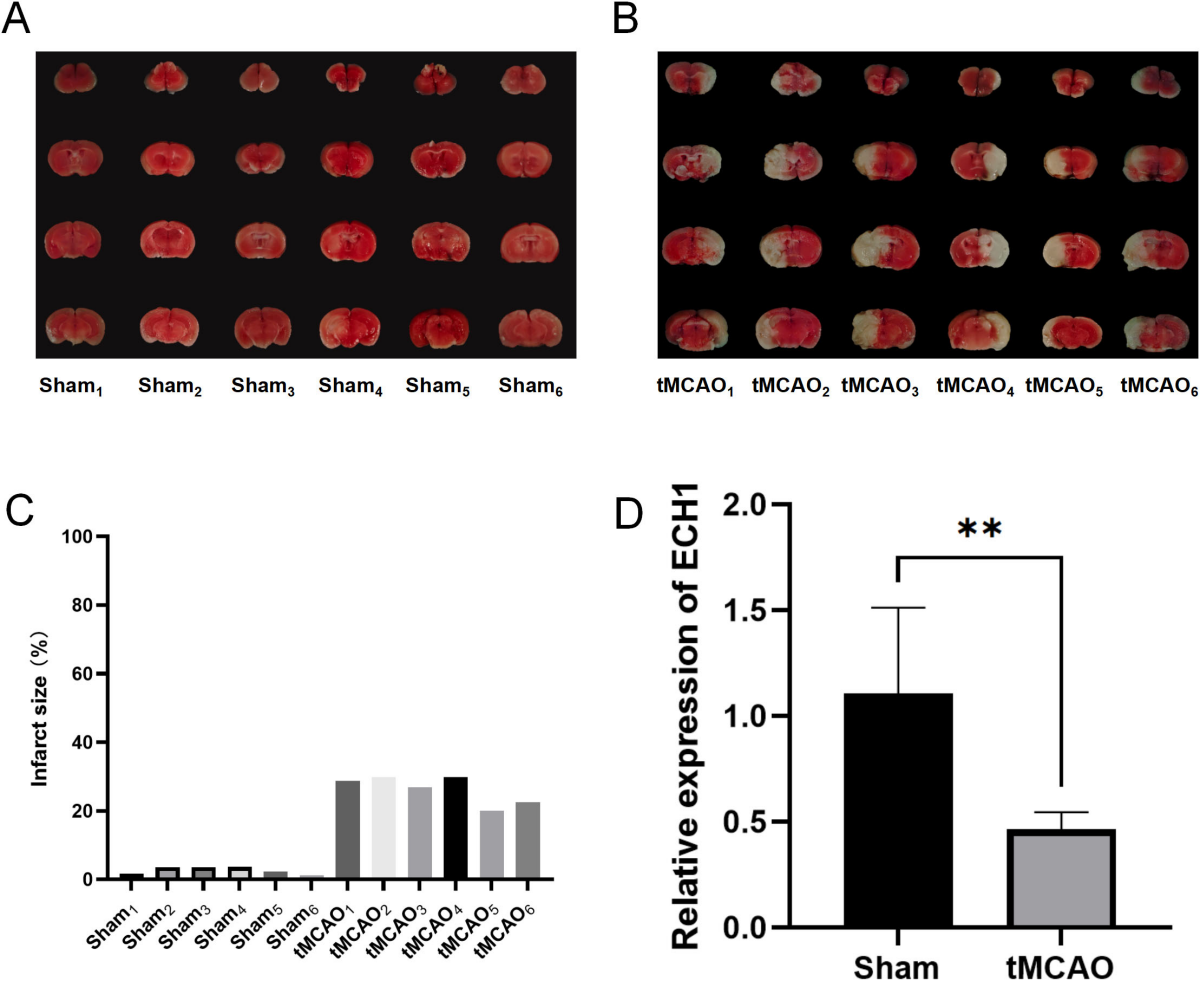
Expression levels of enoyl-coA hydrolase 1 (ECH1) in transient middle cerebral artery occlusion (tMCAO) mice. **(A)** Cerebral infarction volume in the sham group, TTC staining. **(B)** Cerebral infarction volume in the tMCAO group, TTC staining. **(C)** Detection of cerebral infarct volume. **(D)** Relative mRNA expression of ECH1. *n = 6*, two-tailed Student’s *t*-test, **p < 0.01.

To validate the reliability of the previous results, we examined the mRNA levels of ECH1 in the *peripheral blood of* tMCAO mice. First, we evaluated the tMCAO model to ensure successful modeling. TTC staining showed the presence of obvious infarct foci in the tMCAO group compared with the sham group (Figures 6A–C). Subsequently, we examined the expression of ECH1 in the peripheral blood. qPCR results showed that ECH1 mRNA levels was significantly decreased in the tMCAO model (*n* = 6, *P* < 0.05) (Figure 6D). This finding is consistent with the down-regulation of ECH1 gene expression in the peripheral blood of IS patients observed in transcriptome analysis and the association between down-regulation of ECH1 and increased IS risk predicted by Mendelian randomization.

### 2.7 ECH1 is relatively lowly expressed in cerebral ischemic areas in the tMCAO model mice

**FIGURE 7:**
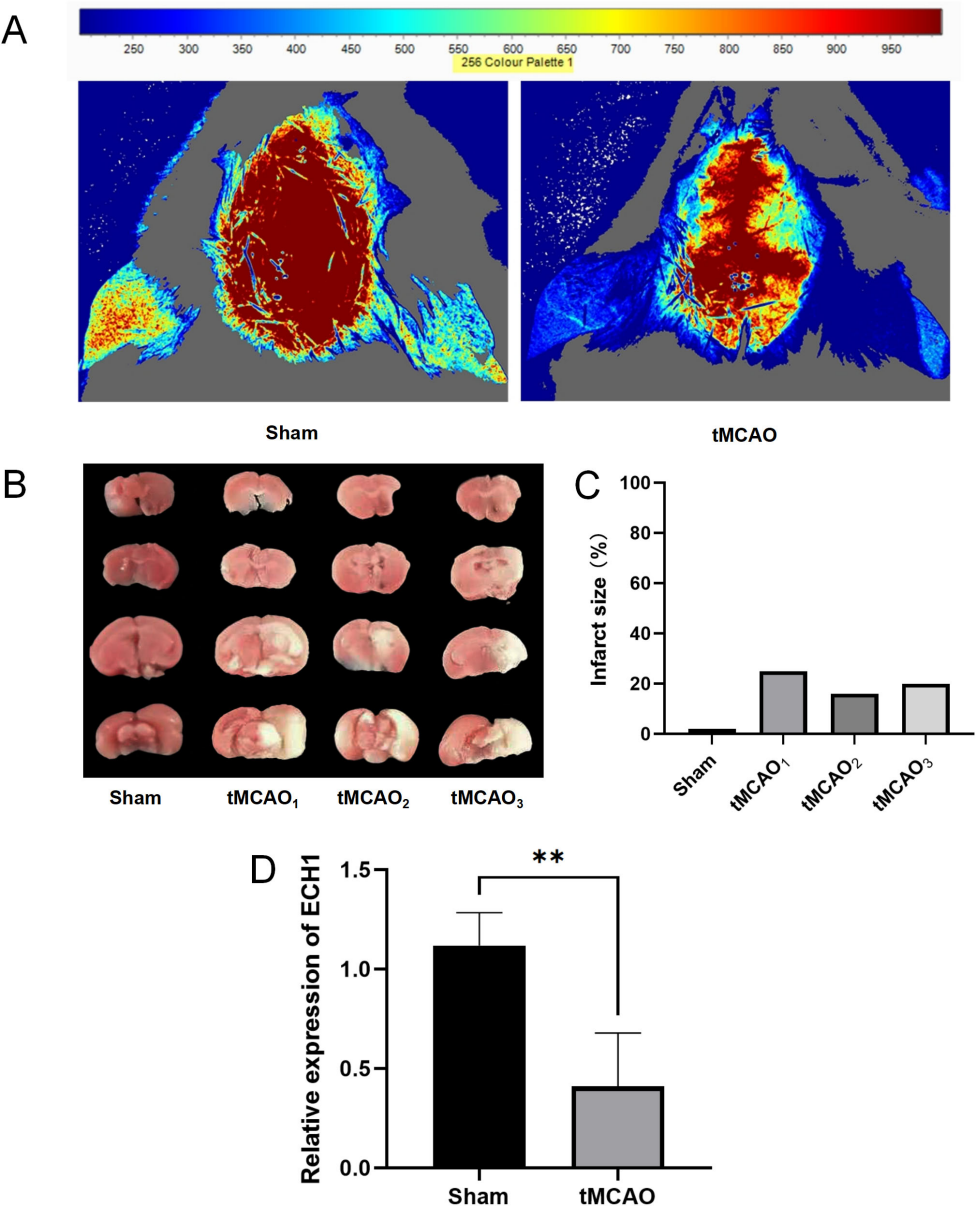
Expression levels of enoyl-coA hydrolase 1 (ECH1) in transient middle cerebral artery occlusion (tMCAO) mice. **(A)** The cerebral blood flow (CBF) was measured by laser scattering. **(B)** The volume of cerebral infarction in mice, TTC Staining. **(C)** Detection of cerebral infarct volume. **(D)** Relative mRNA expression of ECH1. *n = 5*, two-tailed Student’s *t*-test, **p < 0.01.

Given that peripheral changes may reflect brain alterations, we further examined ECH1 mRNA levels in brain tissues. Before this, we confirmed successful tMCAO modeling. Laser diffusion hemodynamic imaging showed significantly reduced blood perfusion in both contralateral and ipsilateral brains of the tMCAO group (Figure 7A), and consistently, TTC staining revealed distinct infarct foci in the tMCAO group (Figures 7B, C). These findings validated model reliability. Subsequently, we detected ECH1 mRNA levels in brain regions via qPCR. Results showed ECH1 was significantly downregulated in ischemic brain tissues of the tMCAO group compared to the sham group (*n* = 6, *P* < 0.05) (Figure 7D), consistent with its mRNA down-regulation in peripheral blood.

## 3 Discussion

Ischemic stroke (IS), which accounts for the majority of strokes, is caused by a blockage of blood flow to the brain and is one of the leading causes of death globally. In clinical practice, the assessment of the severity of brain injury mainly relies on imaging tests. Although peripheral blood biomarkers have shown promising value in aiding diagnosis, they have not yet been fully integrated into the standard clinical metrics (Amini et al., 2023; Harpaz et al., 2020; Jickling and Sharp, 2011). As a hot research topic in recent years, ferroptosis has been found to play an important role in the neurological damage caused by IS, and it is clinically important to explore the diagnostic and therapeutic options for IS based on the ferroptosis perspective (Bu et al., 2021; Fang et al., 2022).

In this study, by screening key ferroptosis genes associated with the pathogenesis of IS, we discovered potential biomarkers and therapeutic targets, with the aim of improving the diagnostic efficacy and therapeutic effectiveness, reducing the degree of neurological damage, and improving the prognosis of patients.

First, through differential expression analysis and machine learning screening, we identified 12 hub differentially expressed genes associated with ferroptosis in the peripheral blood of IS patients. Among these genes, ALOX5 (arachidonic acid 5-lipoxygenase) showed a strong positive correlation with SLC2A3 (solute carrier family 2 member 3), indicating activation of the arachidonic acid metabolic pathway. SLC2A3-mediated glucose uptake can contribute to NADPH production via the pentose phosphate pathway (PPP) (TeSlaa et al., 2023; Wu et al., 2021). Meanwhile, the catalytic activity of ALOX5 depends on reducing equivalents supplied by NADPH (Santha and Vishwanathan, 2022). This metabolic interaction suggests a possible feedback mechanism that may enhance the activation of the arachidonic acid metabolic pathway, though direct regulatory evidence remains to be established.

Subsequently, immune analysis showed that the ratio of monocytes to neutrophils in the peripheral blood of IS patients was accompanied by a marked upregulation of the pro-inflammatory cytokines IFN-α1 and IL-1A. Further, our analyses revealed that these peripheral blood immune alterations correlate notably with core ferroptosis genes (e.g., ECH1). This correlation suggests that core ferroptosis genes may contribute to disrupted peripheral blood immune homeostasis by regulating ferroptotic processes, consistent with prior studies showing that ferroptosis influences immune cell recruitment and activation through DAMP release (Chen et al., 2023; Jost and Höckendorf, 2019; Zeng et al., 2024). Specifically, aberrant expression of these genes may act as a potential upstream driver of monocyte/neutrophil infiltration and pro-inflammatory cytokine upregulation in the peripheral blood of IS patients. This also suggests that targeting these genes could mitigate both ferroptosis-related pathology and peripheral immune dysfunction. Meanwhile, peripheral blood alterations of these 12 molecules in IS patients have no significant gender or age bias, indicating their stability as biomarkers. It is noteworthy that there was a difference between the untreated group (3 h) and the treated group (5 h) (*P* < 0.05) (Supplementary Figure 3), suggesting that treatment may result in an elevated ferroptosis signal due to ischemia-reperfusion injury.

Although all 13 candidate genes showed potential association with IS, Mendelian randomization analysis further revealed a negative causal relationship between peripheral ECH1 and stroke risk. This result suggests that ECH1 may not simply be a concomitant alteration of the disease, but may be involved in the potential pathogenic factors of stroke development. In addition, we also observed a significant down-regulation trend of ECH1 in the brain regions of the tMCAO model mice, a finding that further validates the possible important role of ECH1 in the pathological process of IS.

Enoyl-coA hydrolase 1 is a key enzyme in fatty acid β-oxidation, which encodes mitochondrial short-chain enoyl-CoA hydrolase 1 (Liang et al., 1999). Its down-regulated expression may affect the metabolic pathway of polyunsaturated fatty acids (PUFA), leading to the intracellular accumulation of PUFA (Wang et al., 2025; Zeng et al., 2025). Excessive PUFAs may exacerbate lipid peroxidation through free radical reactions, thereby triggering ferroptosis and ultimately exacerbating neuronal damage after IS (Yang et al., 2016). The consistent expression of ECH1 in brain regions and peripheral blood in this study supports its role as a key molecule in the ferroptosis pathway. Future studies could clarify its specific upstream and downstream mechanisms through functional experiments (e.g., knockdown or overexpression of ECH1).

Notably, there are several limitations in this study that warrant acknowledgment. First, although the present study focused on the role of ECH1 of the ferroptosis pathway in IS, there are other biological functions of this gene independent of ferroptosis, such as its regulatory role in fatty acid metabolism or mitochondrial homeostasis, which may affect IS pathology through non-ferroptosis pathways (Huang et al., 2018; Sakai et al., 2015). Second, our findings only preliminarily establish an association between ECH1 and IS, with no definitive evidence for a causal relationship. Third, as a cross-sectional study conducted at a single time point, it lacks longitudinal tracking of ECH1 dynamics during stroke onset and progression. Fourth, functional validation, including ECH1 knockdown and rescue assays, was not performed, leaving its potential role in IS pathogenesis unsubstantiated. Addressing these limitations in future research will be important to clarify whether ECH1 holds promise as a diagnostic marker and therapeutic target for IS.

## 4 Conclusion

In summary, this study highlights the ferroptosis pathway as a promising avenue for precision therapy in IS. Using the ferroptosis gene set as an entry point, preliminary multidimensional validation links ECH1 to IS, supporting its potential as a candidate peripheral diagnostic indicator and therapeutic target. These findings contribute to our understanding of ferroptosis in IS pathogenesis and provide a foundational basis for exploring diagnostic and therapeutic approaches related to ECH1. Further validation through mechanistic and clinical studies is needed to confirm their clinical applicability and mechanistic relevance.

## 5 Materials and methods

### 5.1 Dataset collection and pre-processing

The datasets GSE16561 and GSE58294 were downloaded from the GEO database. Each dataset was internally normalized using the Limma package of R (version 4.2.3) to reduce batch effects on sequencing depth and to perform log2 transformations on gene expression. The SVA package was used to de-batch genes that coexisted between the two datasets, and PCA plots were used to show before and after differences.

### 5.2 Ferroptosis-related gene collection and Single Sample Gene Set Enrichment Analysis (ssGSEA)

Genes associated with ferroptosis were collected through the FerrDb database^1^. The ssGSEA method in the GSVA package was used to score the expression of ferroptosis-related genes in each sample, and then the differences between the healthy control and IS groups were analyzed.

### 5.3 Differential gene analysis of the ferroptosis gene set

The Limma package was used to analyze the differentially expressed genes in the ferroptosis gene set with Benjamini-Hochberg correction. Absolute log2 fold change ≥ 0.5 and adjusted *p*-value < 0.05 were set as the screening threshold.

### 5.4 Construction of training and validation cohorts via stratified random sampling

Training and validation cohorts were constructed using the createDataPartition function from the caret package. A stratified random sampling approach was employed to allocate 70% and 30% of the dataset to the training and validation subsets, respectively. This ensured proportional representation of class distributions across partitions, maintaining statistical consistency with the original dataset.

### 5.5 Support vector machine and random forest model screening of hub genes

All machine learning analyses were conducted in R (version 4.4.2) using the caret package (version 7.0-1). Support Vector Machine (SVM) models were implemented via the “svmRadial” method, and Random Forest (RF) models via the rf’ method with 1,000 decision trees. Feature selection was performed using Recursive Feature Elimination (RFE) combined with 10-fold cross. Model performance during feature selection was evaluated using classification accuracy and area under the receiver operating characteristic curve (AUC-ROC). Core genes were identified as the intersection of the top-ranked gene sets from both SVM and RF models, enhancing the reliability of feature selection. This consensus gene set was used for all subsequent analyses, ensuring robustness across algorithms.

### 5.6 Diagnosing the discriminative power of hub genes

To validate the diagnostic efficacy of the hub genes identified through machine learning, receiver operating characteristic (ROC) curve analysis was performed using the training cohort to differentiate between ischemic stroke (IS) patients and healthy controls. The area under the curve (AUC) served as the primary metric, with higher values indicating superior discriminative accuracy for potential IS biomarkers. The robustness of these hub genes was further confirmed via identical ROC analysis in an independent validation cohort, ensuring consistent performance across distinct cohorts. All analyses were conducted using the pROC package (version 1.18.5) in R.

### 5.7 Immune infiltration analysis

The CIBERSORT package was used to calculate the relative levels of immune cells and inflammatory factors in each sample as a result of immune infiltration. These results were then compared with key ferroptosis-related genes to identify genes that are closely linked to immune regulation in this process.

### 5.8 Cluster analysis

The core genes with adjusted *p*-value < 0.05 and absolute log2 fold change ≥ 0.5 in different subgroups were selected. The consensus scoring on key genes was obtained by the Consensus Cluster Plus package. Cluster analysis was based on these results, and clinical-related information was compared among different cluster subgroups.

### 5.9 Mendelian randomization analysis

The eQTLGen Consortium contains a full set of cis-eQTLs data of peripheral blood from 31,684 individuals. Statistically significant cis-eQTL were obtained from this database (false discovery rate < 0.05, ± 1 MB from each probe), and cis-eQLT in the ± 100 kb range of the ECH1 genome were extracted. GWAS summary statistics for Stoke were obtained from the FinnGen Consortium. This study included 53,492 patients with stroke and 360,342 controls. The GWAS was adjusted for the following covariates: age, sex, 10 principal components, and genotyping batch.

MR analyses were performed using the TwoSampleMR R package. Prior to MR analyses, low-quality genetic instrumental variables were filtered by several rules. First, we excluded weak-strength SNPs (F-statistic < 10). Then, after harmonizing the exposure and outcome summary data, we selected conditionally independent SNPs without chain disequilibrium (r^2^ < 0.1, based on 1,000 Genomes European reference panel) as instrumental variables. MR estimates for each SNP were calculated using the Wald ratio method, and meta-analysis of multiple SNP estimates was performed with the IVW method as the main method, while sensitivity analysis was performed by MR-Egger, weighted median, simple mode, and Weighted mode. Potential pleiotropy of the association between exposure and outcome was assessed by MR-Egger regression. Heterogeneity of effects between genetic instruments was assessed by Cochran’s Q statistic.

### 5.10 Design of the animal experiments

A total of 8 weeks-old male C57BL/6J mice of specific pathogen-free grade, weighing 23 ± 2 g, were provided by Zhi Shan (Beijing) Health Medical Research Institute Co., Ltd. The experimental animals were housed in a Turnover room at Beijing University of Chinese Medicine, with a 12 h light/dark cycle maintained at 25 ± 1°C and 55 ± 10% humidity. The environment was free from noise interference. This study was approved and supervised by the Animal Ethics Committee of Beijing University of Chinese Medicine, ensuring animal welfare throughout the experimental design and procedures. All experiments were carried out following the guidelines of the Beijing University of Chinese Medicine Committee for animals. Mice were handled and maintained according to the Beijing University guidelines for animal experimentation. Ethics approval number is: BUCM-2024111506-4290. The date of approval is: 2024/11/15.

### 5.11 Middle cerebral artery occlusion (MCAO) mice model

Male C57BL/6J mice (8 weeks old) weighing 25 g (±2 g) were selected and anesthetized with sodium pentobarbital (40 mg/kg). After anesthesia with tribromoethanol, the mice were fixed in the supine position, and the skin was incised along the middle of the neck to isolate the common carotid artery (CCA) on one side, carefully detach the vagus nerve, and temporarily ligate the CCA with a vessel suture. The external carotid artery (ECA) and internal carotid artery (ICA) are separated, two tight sutures are tied distally to the ECA, a small incision is carefully made in the vessel with the aid of an arterial clip, and a spigot is inserted into the ICA from the stump of the ECA until the beginning of the middle cerebral artery (MCA) is occluded, and the tail end of the spigot is exposed outside the body as the wound is sutured, and the spigot is removed by passing it through the tail end one hour later.

### 5.12 Detection of cerebral blood flow (CBF)

The mice were fixed on the stereotaxic device. The skin at the top of the head was routinely sterilized, and then the scalp was incised. The meninges were then separated to completely expose the skull between the coronal suture and the crypt. Then, cortical CBF was assessed by laser scatter flow imaging.

### 5.13 TTC

The C57BL/6J mice were craniotomized 24 h after the completion of modeling, and the brain tissue was extracted and immediately frozen in a −20°C refrigerator for 30 min. Frozen brain tissue is cut into 5–6 coronal slices of approximate thickness and placed in a Petri dish containing 1% TTC solution, ensuring that the TTC solution completely submerges the brain tissue slices, and incubated at 37°C for 30 min, followed by gentle rinsing with PBS. The stained brain tissue was fixed in 4% paraformaldehyde solution for 1 h.

### 5.14 RNA extraction and RT-qPCR

The reagents and primers for RNA extraction and RT-qPCR were obtained from Accurate Biotechnology (Hunan) Co., Ltd., China. Total RNA was extracted from the mouse brain using SteadyPure Universal RNA Extraction Kit (AG21017). cDNA was reverse transcribed from the extracted RNA using Evo M-MLV RT Premix for qPCR (AG11706). The mRNA expression levels were quantified using a real-time fluorescence quantitative PCR analyzer (C6, TargetingOne, China) and a ChamQ Blue Universal SYBR qPCR Master Min (Q312-02). All experimental procedures were performed according to the manufacturer’s protocol. Sangon Biotech ACTB (B661302-0001) was used as an internal reference gene to detect the expression of related mRNAs in brain tissues.

Ech1 primers were customized from Sangon Biotech, and the primers were Ech1-left primer 1 (atggccacctggaacatgag), Ech1-right primer 1 (ctctctcagggtccggaaga).

## Data Availability

Publicly available datasets were analyzed in this study. This data can be found here: https://www.ncbi.nlm.nih.gov/geo/ with accession numbers GSE16561 and GSE58294.

## Footnotes

1
http://www.zhounan.org/ferrdb/

## References

[B1] Amini H. Knepp B. Rodriguez F. Jickling G. C. Hull H. Carmona-Mora P. (2023). Early peripheral blood gene expression associated with good and poor 90-day ischemic stroke outcomes. *J. Neuroinflamm.* 20:13. 10.1186/s12974-022-02680-y 36691064 PMC9869610

[B2] Bu Z.-Q. Yu H.-Y. Wang J. He X. Cui Y.-R. Feng J.-C. (2021). Emerging role of ferroptosis in the pathogenesis of ischemic stroke: A new therapeutic target? *ASN Neuro* 13:17590914211037505. 10.1177/17590914211037505 34463559 PMC8424725

[B3] Chai Z. Zheng J. Shen J. (2024). Mechanism of ferroptosis regulating ischemic stroke and pharmacologically inhibiting ferroptosis in treatment of ischemic stroke. *CNS Neurosci. Ther.* 30:e14865. 10.1111/cns.14865 39042604 PMC11265528

[B4] Chen Y. Fang Z.-M. Yi X. Wei X. Jiang D.-S. (2023). The interaction between ferroptosis and inflammatory signaling pathways. *Cell Death Dis.* 14:205. 10.1038/s41419-023-05716-0 36944609 PMC10030804

[B5] Dixon S. J. Lemberg K. M. Lamprecht M. R. Skouta R. Zaitsev E. M. Gleason C. E. (2012). Ferroptosis: An iron-dependent form of nonapoptotic cell death. *Cell* 149 1060–1072. 10.1016/j.cell.2012.03.042 22632970 PMC3367386

[B6] Fan W. Zheng J. Jiang F. (2025). Analysis of ferroptosis-related genes in cerebral ischemic stroke via immune infiltration and single-cell RNA-Sequencing. *BMC Med. Genom.* 18:31. 10.1186/s12920-025-02098-4 39934808 PMC11817822

[B7] Fang X.-L. Ding S.-Y. Du X.-Z. Wang J.-H. Li X.-L. (2022). Ferroptosis-A novel mechanism with multifaceted actions on stroke. *Front. Neurol.* 13:881809. 10.3389/fneur.2022.881809 35481263 PMC9035991

[B8] Gowtham A. Chauhan C. Rahi V. Kaundal R. K. (2024). An update on the role of ferroptosis in ischemic stroke: From molecular pathways to neuroprotection. *Exp. Opin. Ther. Targets* 28 1149–1175. 10.1080/14728222.2024.2446319 39710973

[B9] Harpaz D. Seet R. C. S. Marks R. S. Tok A. I. Y. (2020). Blood-Based biomarkers are associated with different ischemic stroke mechanisms and enable rapid classification between cardioembolic and atherosclerosis etiologies. *Diagnostics* 10:804. 10.3390/diagnostics10100804 33050269 PMC7600601

[B10] He Y. Wang J. Ying C. Xu K. L. Luo J. Wang B. (2024). The interplay between ferroptosis and inflammation: Therapeutic implications for cerebral ischemia-reperfusion. *Front. Immunol.* 15:1482386. 10.3389/fimmu.2024.1482386 39582857 PMC11583640

[B11] Hu X. Bao Y. Li M. Zhang W. Chen C. (2024). The role of ferroptosis and its mechanism in ischemic stroke. *Exp. Neurol.* 372:114630. 10.1016/j.expneurol.2023.114630 38056585

[B12] Huang D. Liu B. Huang K. Huang K. (2018). Enoyl coenzyme a hydratase 1 protects against high-fat-diet-induced hepatic steatosis and insulin resistance. *Biochem. Biophys. Res. Commun.* 499 403–409. 10.1016/j.bbrc.2018.03.052 29526751

[B13] Jickling G. C. Sharp F. R. (2011). Blood biomarkers of ischemic stroke. *Neurotherapeutics* 8 349–360. 10.1007/s13311-011-0050-4 21671123 PMC3250275

[B14] Jost P. J. Höckendorf U. (2019). Necroinflammation emerges as a key regulator of hematopoiesis in health and disease. *Cell Death Differ.* 26 53–67. 10.1038/s41418-018-0194-4 30242210 PMC6294770

[B15] Liang X. Zhu D. Schulz H. (1999). Δ3,5,7,Δ2,4,6-Trienoyl-CoA isomerase, a novel enzyme that functions in the β-oxidation of polyunsaturated fatty acids with conjugated double bonds. *J. Biol. Chem.* 274 13830–13835. 10.1074/jbc.274.20.13830 10318788

[B16] Liu C. Sui H. Li Z. Sun Z. Li C. Chen G. (2025). THBS1 in macrophage-derived exosomes exacerbates cerebral ischemia-reperfusion injury by inducing ferroptosis in endothelial cells. *J. Neuroinflamm.* 22:48. 10.1186/s12974-025-03382-x 39994679 PMC11854006

[B17] Liu C. P. Zheng S. Zhang P. Chen G.-H. Zhang Y.-Y. Sun H.-L. (2025). Decreased serum SLC7A11 and GPX4 levels may reflect disease severity of acute ischaemic stroke. *Ann. Clin. Biochem.* 62 191–201. 10.1177/00045632241305927 39632577

[B18] Liu D. Yang S. Yu S. (2024). Interactions between ferroptosis and oxidative stress in ischemic stroke. *Antioxidants* 13:1329. 10.3390/antiox13111329 39594471 PMC11591163

[B19] Lu J. Xu F. Lu H. (2020). LncRNA PVT1 regulates ferroptosis through miR-214-mediated TFR1 and P53. *Life Sci.* 260:118305. 10.1016/j.lfs.2020.118305 32827544

[B20] Martin S. S. Aday A. W. Allen N. B. Almarzooq Z. I. Anderson C. A. M. Arora P. (2025). 2025 heart disease and stroke statistics: A report of us and global data from the american heart association. *Circulation* 151 e41–e660. 10.1161/CIR.0000000000001303 39866113 PMC12256702

[B21] Sakai C. Yamaguchi S. Sasaki M. Miyamoto Y. Matsushima Y. Goto Y. (2015). ECHS1 mutations cause combined respiratory chain deficiency resulting in leigh syndrome. *Hum. Mutat.* 36 232–239. 10.1002/humu.22730 25393721

[B22] Santha S. S. R. Vishwanathan A. S. (2022). Mechanistic insights into 5-Lipoxygenase inhibition by pyocyanin: A molecular docking and molecular dynamics study. *J. Biomol. Struct. Dyn.* 40 9752–9760. 10.1080/07391102.2021.1934543 34143945

[B23] TeSlaa T. Ralser M. Fan J. Rabinowitz J. D. (2023). The pentose phosphate pathway in health and disease. *Nat. Metab.* 5 1275–1289. 10.1038/s42255-023-00863-2 37612403 PMC11251397

[B24] Tuo Q.-Z. Lei P. (2024). Ferroptosis in ischemic stroke: Animal models and mechanisms. *Zool. Res.* 45 1235–1248. 10.24272/j.issn.2095-8137.2024.239 39397243 PMC11668946

[B25] Wang Y. Hu M. Cao J. Wang F. Han J. R. Wu T. W. (2025). ACSL4 and polyunsaturated lipids support metastatic extravasation and colonization. *Cell* 188 412–429.e27. 10.1016/j.cell.2024.10.047. 39591965

[B26] Wang Y. Wu S. Li Q. Sun H. Wang H. (2023). Pharmacological inhibition of ferroptosis as a therapeutic target for neurodegenerative diseases and strokes. *Adv. Sci.* 10:e2300325. 10.1002/advs.202300325 37341302 PMC10460905

[B27] Wu D. Harrison D. L. Szasz T. Yeh C.-F. Shentu T.-P. Meliton A. (2021). Single-Cell metabolic imaging reveals a SLC2A3-Dependent glycolytic burst in motile endothelial cells. *Nat. Metab.* 3 714–727. 10.1038/s42255-021-00390-y 34031595 PMC8362837

[B28] Yang W. S. Kim K. J. Gaschler M. M. Patel M. Shchepinov M. S. Stockwell B. R. (2016). Peroxidation of polyunsaturated fatty acids by lipoxygenases drives ferroptosis. *Proc. Natl. Acad. Sci. U. S. A.* 113 E4966–E4975. 10.1073/pnas.1603244113 27506793 PMC5003261

[B29] Yeh S.-J. Chen C.-H. Lin Y.-H. Tsai L.-K. Lee C.-W. Tang S.-C. (2023). Association of ferroptosis with severity and outcomes in acute ischemic stroke patients undergoing endovascular thrombectomy: A case-control study. *Mol. Neurobiol.* 60 5902–5914. 10.1007/s12035-023-03448-y 37357230

[B30] Zeng L. Yang K. Yu G. Hao W. Zhu X. Ge A. (2024). Advances in research on immunocyte iron metabolism, ferroptosis, and their regulatory roles in autoimmune and autoinflammatory diseases. *Cell Death Dis.* 15:481. 10.1038/s41419-024-06807-2 38965216 PMC11224426

[B31] Zeng Y. Zhao L. Zeng K. Zhan Z. Zhan Z. Li S. (2025). TRAF3 loss protects glioblastoma cells from lipid peroxidation and immune elimination via dysregulated lipid metabolism. *J. Clin. Invest.* 135:e178550. 10.1172/JCI178550 39932808 PMC11957706

[B32] Zhang Y. Lu X. Tai B. Li W. Li T. (2021). Ferroptosis and its multifaceted roles in cerebral stroke. *Front. Cell Neurosci.* 15:615372. 10.3389/fncel.2021.615372 34149358 PMC8209298

[B33] Zhang Y. Zhang Y. Yao R. He X. Zhao L. Zuo X. (2022). Ferroptosis-Related differentially expressed genes serve as new biomarkers in ischemic stroke and identification of therapeutic drugs. *Front. Nutr.* 9:1010918. 10.3389/fnut.2022.1010918 36438734 PMC9686348

