# Supplementary material for: Exploring ischemic stroke based on the ferroptosis perspective: ECH1 may serve as a new biomarker and therapeutic target
Source: Front Neurosci. 2025 Aug 22;19:1622760. doi: 10.3389/fnins.2025.1622760 (PMC12411446; doi:10.3389/fnins.2025.1622760)
Supplement: Supplementary file 1 [file Data_Sheet_1.pdf]

Supplemental Materials

Figure S1

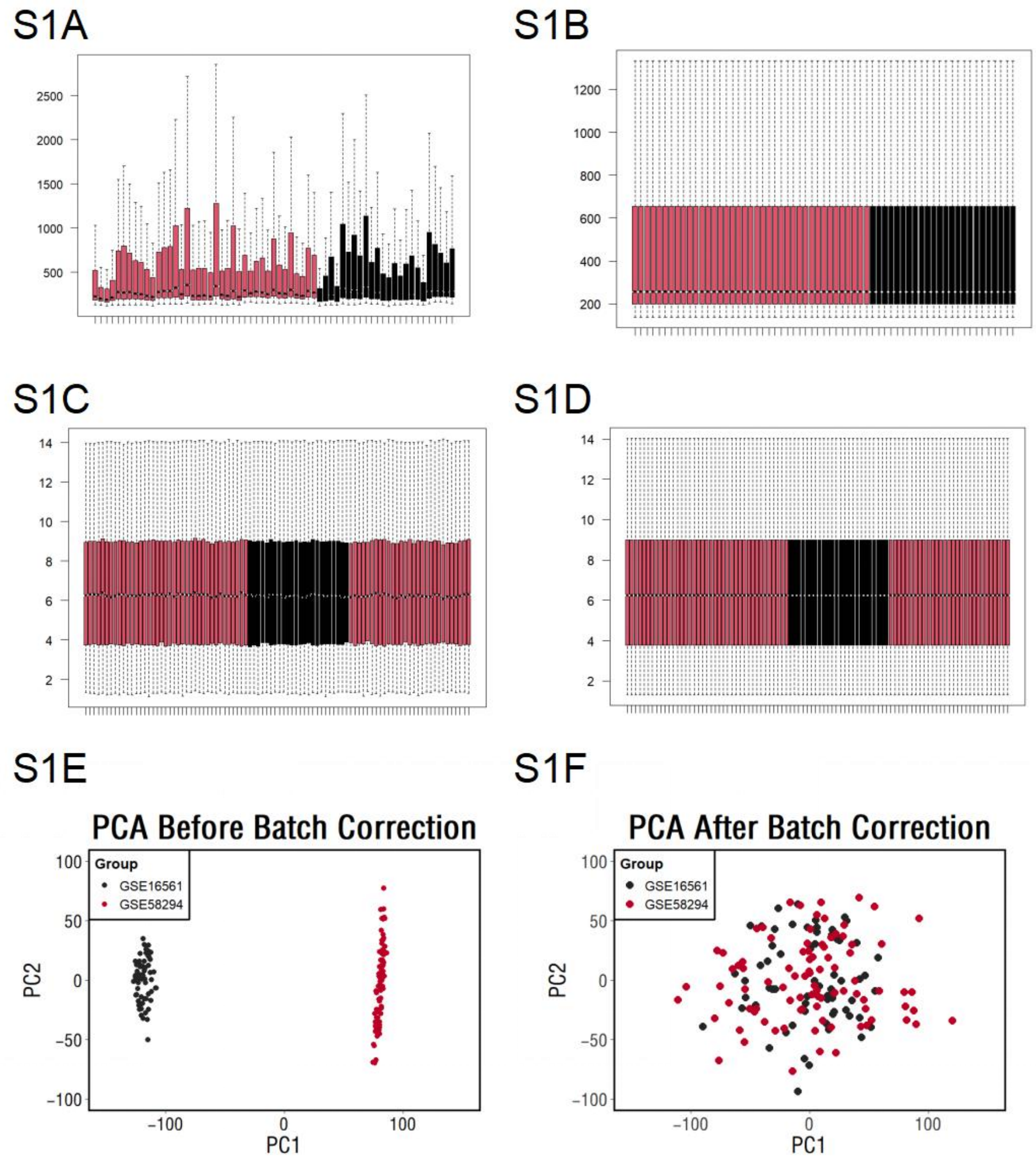

Figure S1. Batch Effect Correction Visualization

(S1A, S1C) Gene expression distribution in the GSE16561 (S1A) and GSE58294 (S1C) datasets before normalization. (S1B, S1D) Gene expression distribution in the GSE16561 (S1B) and GSE58294 (S1D) datasets after normalization. (S1E) PCA visualization of the dataset before batch effect removal. (S1F) PCA visualization of the dataset after batch effect removal.

Figure S2

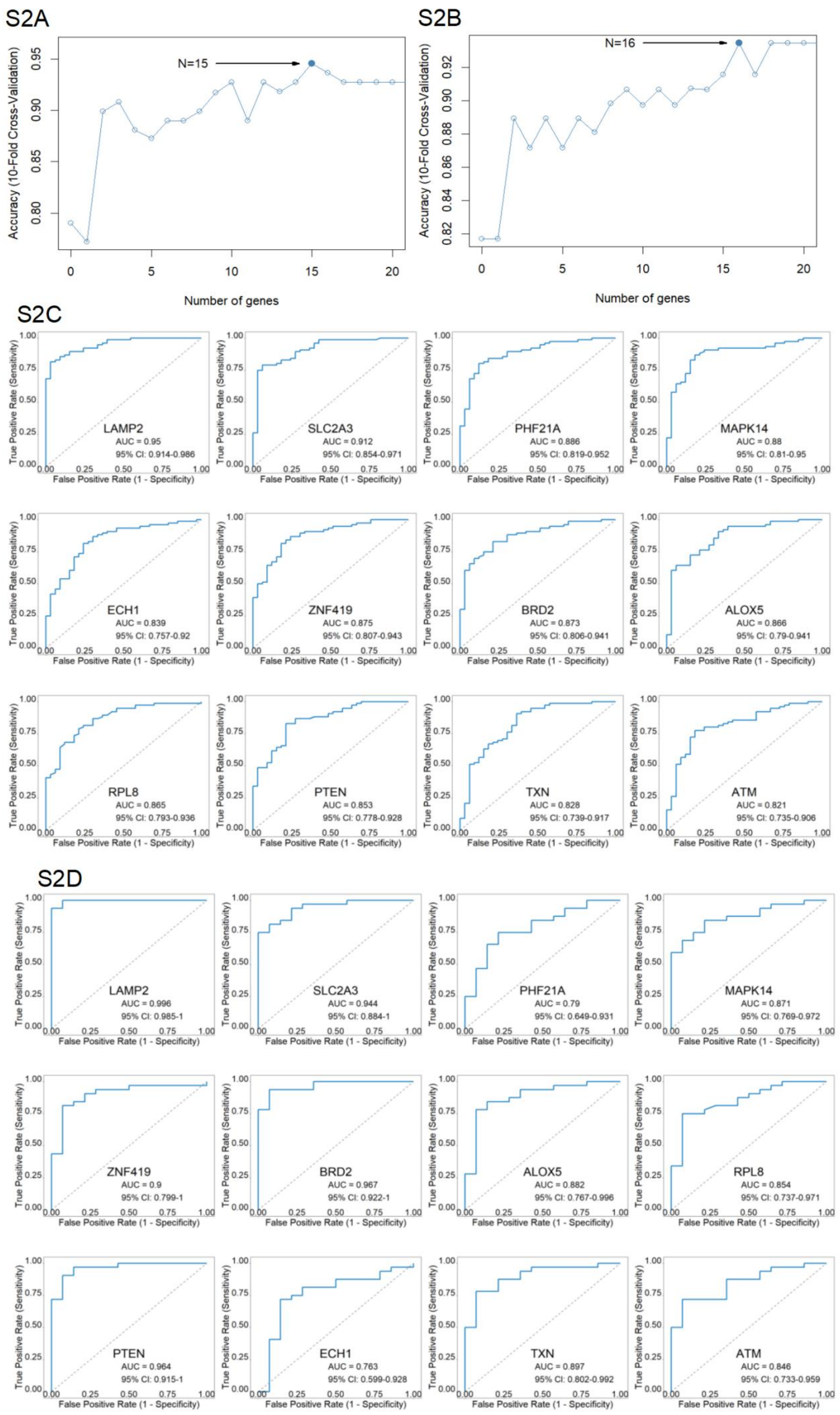

Figure S2. Feature selection and AUC-ROC curve.

(S2A) The relationship between the number of feature genes and model accuracy via the SVM algorithm. (S2B) The relationship between the number of feature genes and model accuracy via the RF algorithm. (S2C) Hub genes in the training cohort were analyzed using ROC curves. (S2D). Hub genes in the testing cohort were analyzed using ROC curves.

Figure S3

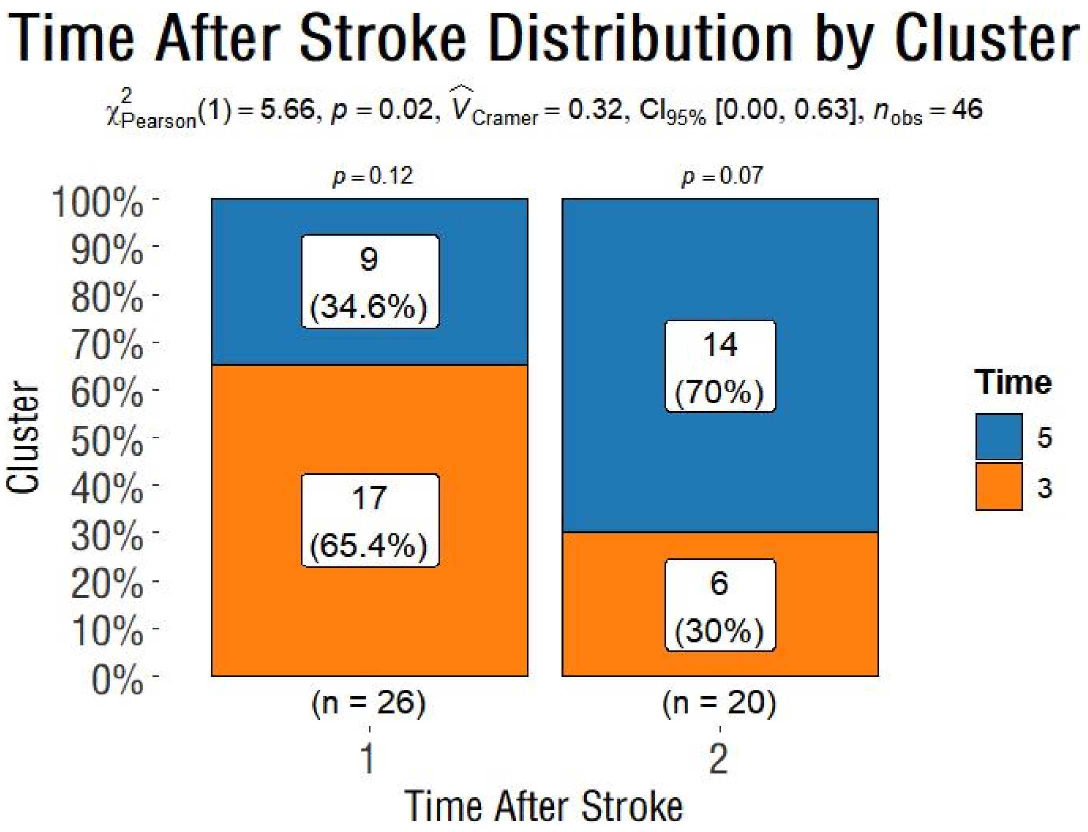

Figure S3. Statistical analysis of stroke onset time differences between two specific subgroups.

This figure supplements Figure 4D by highlighting the statistically significant differences in stroke onset times between two specific subgroups.

Figure S4

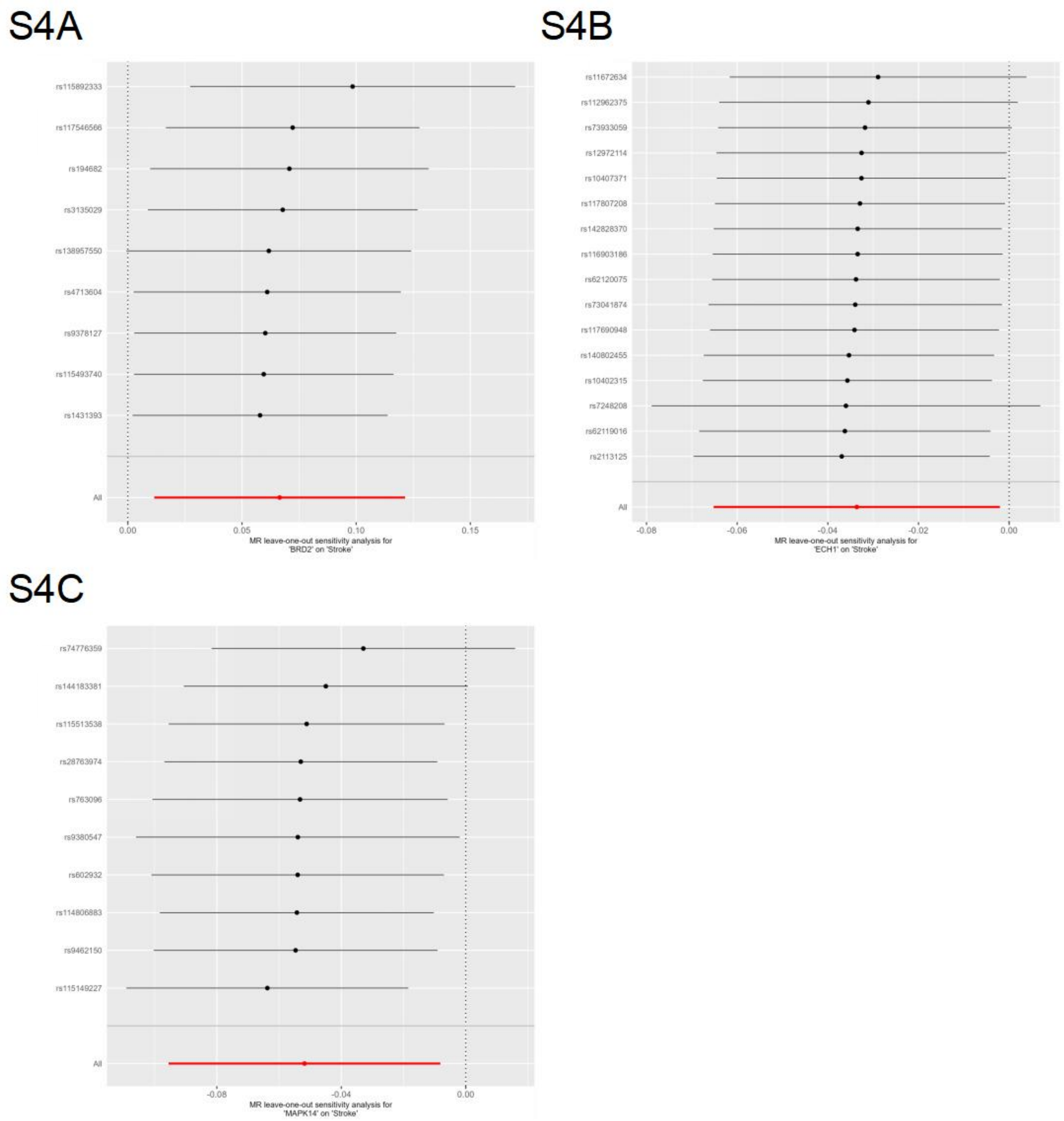

Figure S4. Results of the leave-one-out analysis.

Mendelian Randomization Leave-One-Out Sensitivity Analysis for Genes BRD2 (S4A), ECH1 (S4B), and MAPK14 (S4C) in Relation to Stroke

Figure S5

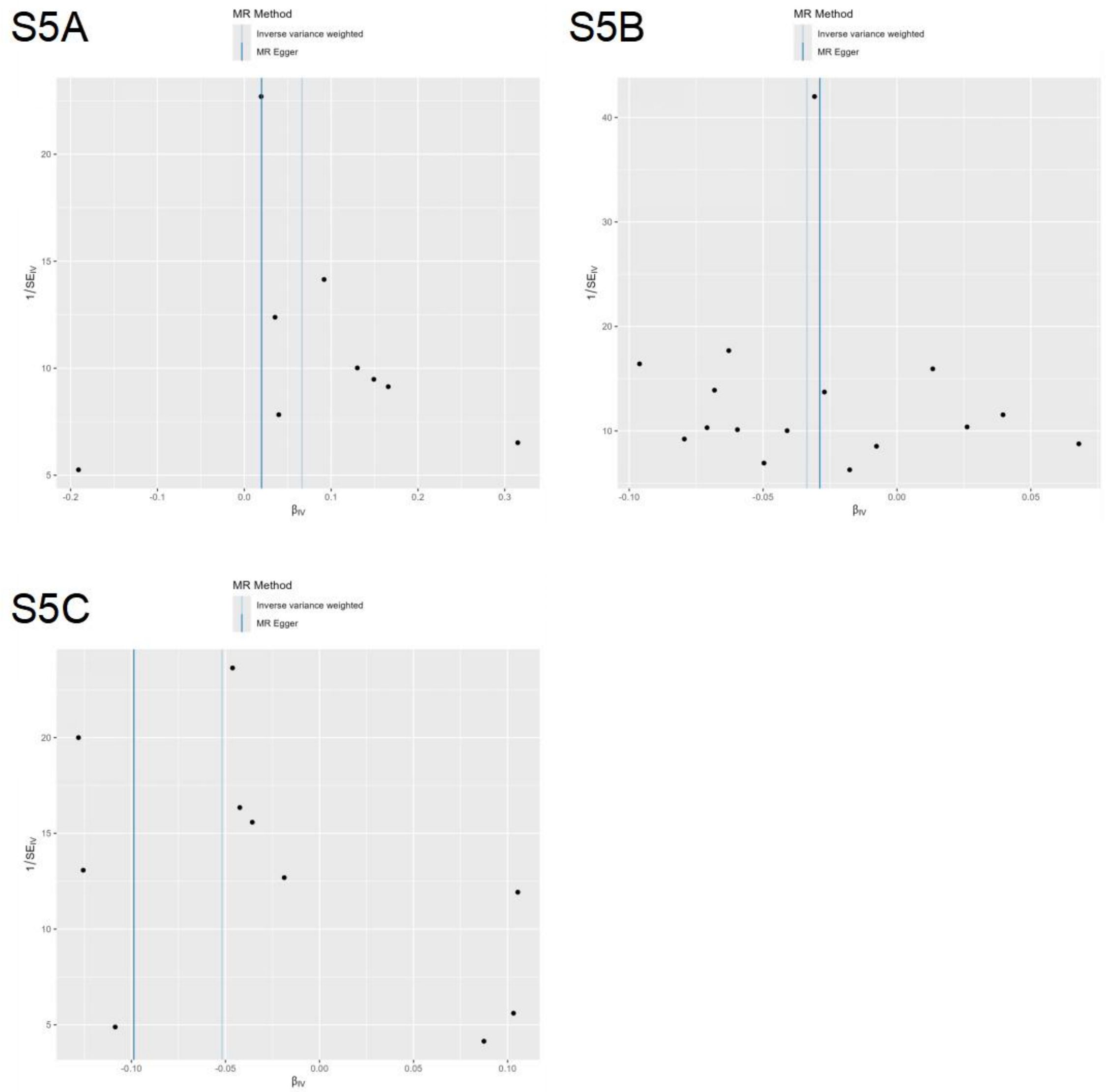

Figure S5. Results of the single-SNP analysis.

Mendelian Randomization Leave-One-Out Sensitivity Analysis for Genes BRD2 (S5A), ECH1 (S5B), and MAPK14 (S5C) in Relation to Stroke
